# PatientVOICE: Development of a Preparatory, Pre-Chemotherapy Online Communication Tool for Older Patients With Cancer

**DOI:** 10.2196/resprot.6979

**Published:** 2017-05-10

**Authors:** Sandra van Dulmen, Jeanine A Driesenaar, Julia CM van Weert, Mara van Osch, Janneke Noordman

**Affiliations:** ^1^ Netherlands Institute for Health Services Research (NIVEL) Utrecht Netherlands; ^2^ Department of Primary and Community Care Radboud University Medical Center Nijmegen Netherlands; ^3^ Faculty of Health Sciences University College of Southeast Norway Drammen Norway; ^4^ Amsterdam School of Communication Research (AScoR) Amsterdam Netherlands

**Keywords:** intervention mapping, chemotherapy, online intervention, communication, patient participation, question prompt sheet, elderly

## Abstract

**Background:**

Good communication around cancer treatment is essential in helping patients cope with their disease and related care, especially when this information is tailored to one’s needs. Despite its importance, communication is often complex, in particular in older patients (aged 65 years or older). In addition to the age-related deterioration in information and memory processing older patients experience, communication is also complicated by their required yet often unmet role of being an active, participatory patient. Older patients rarely express their informational needs and their contributions to consultations are often limited. Therefore, older patients with cancer need to be prepared to participate more actively in their care and treatment.

**Objective:**

The objective of this paper was to report the development of PatientVOICE, an online, preparatory tool with audio facility aimed to enhance the participation of older patients during educational nursing encounters preceding chemotherapy and to improve their information recall.

**Methods:**

PatientVOICE was developed by applying the following 6 steps of the intervention mapping framework that involved both patients and nurses: (1) needs assessment, (2) specifying determinants and change objectives, (3) reviewing and selecting theoretical methods and practical strategies, (4) developing intervention components, (5) designing adoption and implementation, and (6) making an evaluation plan.

**Results:**

A careful execution of these consecutive steps resulted in the ready-to-use preparatory website. PatientVOICE provides pre-visit information about chemotherapy (ie, medical information, side effects, and recommendations of dealing with side effects), information about the educational nursing visit preceding chemotherapy (ie, aim, structure, and recommendations for preparation), techniques to improve patients’ communication skills using a question prompt sheet (QPS) and video-modeling examples showing “best practices”, and the opportunity to upload and listen back to an audio recording of a patient’s own nursing visit.

**Conclusions:**

The development process resulted in PatientVOICE, a multi-component online intervention targeted to older patients with cancer. PatientVOICE contains information about the treatment as well as information about the role of the patient during treatment. Using different methods (QPS and audio facility), we hope to support these patients during their treatment. In the future, the utility and usability of this complex intervention will be evaluated in a group of older patients who receive or have received chemotherapy.

## Introduction

More than 60% of all cancer patients are aged 65 years and above and it is expected that this number will increase due to the aging population [1]. Older patients with cancer are confronted with complex information about treatments, like chemotherapy, that impose high demands on their emotional and cognitive abilities. Communicating with their healthcare provider is even more challenging due to older patients’ deterioration in cognitive (eg, memory, information processing), psychological (eg, resilience), physical (eg, hearing problems, multimorbidity), and social (eg, network, activities) functioning [2,3]. Indeed, during educational nursing encounters preceding chemotherapy, older patients only actively reproduce (recall) less than one fourth of the recommendations given on handling side effects [4].

Effective communication surrounding cancer treatment is essential as this can support patients in coping with their disease, treatment, and side effects [4-7]. This is especially true when the provided information is tailored to the patient’s informational needs [8]. Providing tailored information not only requires advanced communication skills of caregivers, but also asks for more active, informed, and participatory patients [9-11]. To date, the conversational contribution of patients with cancer, and older patients in particular, is rather limited [12-15]. Older patients rarely express their informational needs or preferences [15,16], perceive several barriers to actively participate during the consultation, and lack the skills needed to obtain relevant information [17]. Despite having different needs with respect to information provision than younger patients [5,15,18,19], they equally value discussing realistic expectations and information about dealing with their treatment options and the corresponding side effects in daily life [6]. Older patients, therefore, not only need to be supported during, but also in preparation and/or after their communication with care providers about their treatment. As elderly do not form a homogenous population, attention is warranted for “what works for whom.”

To this purpose, we restructured educational nursing encounters preceding chemotherapy and developed a preparatory paper brochure for older patients in the oncology departments of several Dutch hospitals [7]. The brochure “Talking about chemotherapy” (in Dutch: “In gesprek over chemotherapie”) contained information about the nursing encounter’s aim and topics and a question prompt sheet (QPS). The QPS was a list of statements that a patient could indicate which topics he or she would like to talk about during the encounter. In comparison with a control group, intervention group nurses spoke more about realistic expectations, reduced the amount of information in concordance with the patients’ needs, and their patients asked more questions [7]. Patients’ information recall, however, hardly increased. One possible explanation for this was preparation, the counseling itself was not yet sufficiently tailored to their information needs, or there is a limit to what one can remember from a consultation [20].

The Internet is an important source of information and support for both cancer patients and their relatives [21], including older cancer patients [22]. Older people increasingly use the Internet, with more than half of the elderly aged between 65 and 75 years using it daily [23]. Internet or computer use even appears to be more predictive for using (online) tools than age [3,24,25]. A recent study found that older cancer patients evaluated Web-based health information tools to be very useful and that they were willing to use these kinds of tools [26]. A literature review revealed that online health information tools seem promising to facilitate immediate, intermediate, and long-term outcomes in older patients, including clinical outcomes such as blood pressure, hemoglobin, and cholesterol levels [27]. Aspects such as the burden and availability of (older) end-users and financial means must be taken into account when designing online tools together with patients (co-creation) [28]. Other challenges to consider are the funding of active technology companies and the time it takes to process the results of shorter development cycles.

This paper describes the structured development of PatientVOICE, an elaborated, online version of the paper brochure “Talking about chemotherapy” where older patients with cancer can prepare themselves on the educational nursing encounter preceding chemotherapy. PatientVOICE was built around effective facilities (QPS, audio recordings, video modeling, and preparatory information) known to enhance patient engagement and information recall, and to tailoring nursing information to personal circumstances and the information and emotional needs of the elderly. The results of the evaluation of PatientVOICE will be reported in another paper.

## Methods

### The Intervention Mapping Framework

The 6 steps of the intervention mapping framework were followed to systematically develop PatientVOICE [29] (Table 1). The intervention mapping framework integrates input from the “target group” (ie, older patients) with theoretical and empirical evidence and largely overlaps with the framework of the Medical Research Council for developing complex interventions (ie, interventions with several interacting components) [30]. Intervention mapping describes a process consisting of the following 6 consecutive steps: (1) assessing needs of the target group to identify the problem, (2) specifying determinants and change objectives, (3) reviewing and selecting theoretical methods and practical strategies, (4) developing intervention components, (5) designing an adoption and implementation plan, and (6) making an evaluation plan [29]. Several electronic health (eHealth) programs have been developed using the intervention mapping framework [31-33].

**Table 1:** The intervention mapping framework applied to the development of PatientVOICE.

Step(s)	Description	Task(s)
1	Needs assessment	Assessing patients' needs regarding the nursing encounter preceding chemotherapy and preparing for chemotherapy
		Evaluation of the brochure “Talking about chemotherapy”
2 and 3	Specifying determinants, objectives, theoretical methods, and practical strategies	Specifying determinants and change objectives
		Reviewing scientific literature to identify practical strategies and techniques
4	Intervention development	Development of the intervention prototype PatientVOICE
		Usability testing by patients with cancer and the elderly using a think-aloud procedure
		Judgment of the website by software experts according to 20 heuristics relevant for older Web users
		Adaptation of the prototype on the basis of usability tests
5	Adoption and implementation plan	Invitation of hospitals for participation and to contribute to the development of PatientVOICE
		Creating a support base for the intervention in hospitals
6	Evaluation plan	Describing the study design, procedure, and methods for the evaluation of the intervention

### Step 1: Needs Assessment

The first step in the intervention mapping framework is the needs assessment. Assessing patients’ needs allows for the identification of important topics and preferences that should be integrated into the online tool PatientVOICE. For this needs assessment, we invited oncology nurses and patients treated with chemotherapy from 3 hospitals that consented to participate in this study.

Older patients (65 years or older) that recently had an educational nursing encounter preceding chemotherapy were invited by their oncology nurse for an interview to evaluate the “Talking about chemotherapy” brochure. A total of 10 patients participated, of which 3 (30%, 3/10) were female with a mean age of 76.6 years (range 67 to 83), and 7 (70%, 7/10) were men with a mean age of 72.3 years (range 66 to 90). Of the patients, 3 (30%, 3/10) were accompanied by a spouse.

The content of the brochure is summarized in Multimedia Appendix 1. As this brochure was used as the starting point of the online tool, it was important to identify its strengths and the components that could be improved. In addition, patients were asked about what they thought was important in the encounter preceding chemotherapy and in preparing for chemotherapy. In each hospital that was willing to participate, a coordinator was appointed that became part of the project team and facilitated the contact with the 10 oncology nurses that we interviewed to evaluate the brochure and to identify additional points for improvement. All of the nurses were female with a mean age of 50.0 years (range 37 to 62). The 10 nurses were informed about the study through a presentation by the researcher at their hospital and accepted the invitation for the interview.

### Steps 2 and 3: Specifying Determinants, Objectives, Theoretical Methods, and Practical Strategies

The aim of the second step was to specify determinants and objectives to determine what behaviors or factors could be influenced in order to reach the intended intervention goal. In step 3, we reviewed the scientific literature to identify practical strategies that could be applied to the intervention. It was important that these strategies corresponded with the determinants and change objectives.

### Step 4: Intervention Development

In step 4, the components of the intervention were developed with input from the outcomes of the previous steps to inform the prototype of PatientVOICE. To evaluate and improve the prototype, usability tests were performed with 5 older patients with cancer and 3 older adults without cancer via a “think-aloud procedure”. For this procedure, the participants performed practical tasks using the website while describing what they were doing and expressing what thoughts came to mind. The expressions were audio recorded. In addition, 2 software experts judged the website according to 20 heuristics relevant for older Web users [34].

### Step 5: Adoption and Implementation

The aim of step 5 was to enable and organize the adoption and implementation of the intervention. We approached 7 hospitals to participate, to contribute to the development of PatientVOICE, and to create a support base for the intervention. As indicated in step 1, oncology nurses were part of the project from the start and acted as coordinators in their respective hospitals.

### Step 6: Evaluation Plan

The final step of the intervention mapping framework consisted of a plan to evaluate the feasibility and user-friendliness of the intervention on feasibility. The evaluation plan describes the study design and methods used. Outcome measures that corresponded with the objectives of our intervention were specified.

## Results

### Step 1: Needs Assessment

#### Brochure Evaluation

Of the evaluating patients, 7 (70%, 7/10) were familiar with the brochure, 3 (30%, 3/10) only read the brochure before the encounter, and 4 (40%, 4/10) also filled in the QPS. In addition, 2 (20%, 2/10) patients had not previously received the brochure and 1 (10%, 1/10) patient could not remember it precisely. Patients that did not complete the QPS gave the following reasons: one patient did not consider it useful because he already had the information from his wife and relatives, one patient had been treated previously with chemotherapy and was already familiar with it, and one patient answered that she would hear the information during the encounter.

However, most of the patients appreciated the brochure because it introduced the issues that were going to be addressed during the consultation. Patients considered it helpful in deciding what issues were important to them and found it supported them in asking questions. Although few patients wrote notes in the brochure, some wrote their questions in a notebook or made notes about the information provided by the healthcare provider, while others kept a diary. The nurses observed that most patients used the brochure and they considered the information clear. They also found the brochure to be a valuable preparation tool because it informed the patients about what to expect (ie, the aim and structure of the nursing encounter). Furthermore, some nurses indicated that their patients asked more questions and that the consultation was more focused when patients used the brochure, though other nurses had no experience with patients being hindered in asking questions in general. Some nurses found the statement in the QPS “What my companions can do to support me” difficult to discuss because they did not know how to advise patients in this matter and found it difficult to predict what kind of support a particular patient and his/her companions would need.

#### Chemotherapy Preparation Needs

In general, many patients valued preparatory information about side effects, the practical consequences of those side effects, hygienic-related measures, as well as how to handle things at home. They also valued information about when and how to contact the hospital or nurse.

Patients mentioned that they would appreciate a picture of the outpatient treatment center where they will be treated to visualize the facility and set-up beforehand. Patients also preferred information about which oncology health professionals were going to be present when they were receiving care.

The overload of, and sometimes, contradictory information that patients received from different sources (eg, family and the Internet) upset some patients even though not all of the information was equally relevant to them. Therefore, it is important for patients to know what information applies to them and what information does not.

While they found the brochure useful, some patients said that one just cannot prepare for chemotherapy; it just happens to you and you have to cope with it. Other patients searched for additional information about the treatment and what to expect, for example, via the Internet or patient organizations. Often, patients’ spouses and children looked for information on the Internet as well.

All patients said that they did not attend the nursing encounter on their own, rather were accompanied by a spouse or companion.

#### Suggestions for Information Improvement

Many patients mentioned that they did not fully understand the medication list that was part of the treatment protocol or the list with additional medications to suppress side effects that was provided by the hospital. Patients preferred a clearer structure and overview of medications. Furthermore, half the patients wanted more information about how and when to use the medication. The nurses also agreed that the medication scheme should be made clearer. Some patients also indicated that they would like more specific information about the effects of the medication on their body.

#### Presentation of Information

In general, patients expressed that information needs to be clearly, briefly, and orderly presented and difficult terminology has to be defined or avoided if possible.

#### Implications for the Online Tool

The results of the interviews were used as input for the website. As the brochure was evaluated very positively, most information was transmitted with minor adaptations. Furthermore, the information on the website must be practical, succinct, and not disease specific. One topic that was brought forward was that it was important to state on the website that treatment is personalized, different for every patient, and that one shouldn't compare situations. As patients’ companions are also involved in gathering information about chemotherapy, the website must be accessible for them as well.

### Step 2 and 3: Specifying Determinants, Change Objectives, Theoretical Methods, and Practical Strategies

The objectives of the intervention are (1) to enhance patient participation during educational nursing encounters preceding chemotherapy; and (2) to improve older patients’ information recall. From the literature, it is known that the following techniques are especially effective in promoting participation during medical visits and patients’ recall: (1) pre-counseling preparatory information, (2) QPSs, (3) video modeling, and (4) consultation audio recordings [11,35]. Therefore, these techniques were included in PatientVOICE.

#### Pre-Counseling Preparatory Information

Preparing patients for upcoming consultations appears to have added value [12,36,37]. For instance, Albada and colleagues developed a tailored website that provides information regarding counselees’ pre-visit needs (eg, the procedure of genetic counseling) and a QPS [31]. Results demonstrated that pre-visit (online) education about breast cancer genetic counseling improved counselees’ information recall and knowledge. In addition, the informational needs of prepared counselees were more addressed by the caregiver and counselees became more assertive by sharing their agenda, directing the communication, and checking for understanding [38].

To prepare patients for the nursing encounter and chemotherapy, PatientVOICE provides information about these topics. This information matches with patients’ needs, as assessed in the needs assessment. Information on the website about the nursing encounter includes the aim and structure of the encounter, preparation of the encounter, taking a companion to the encounter, and expressing needs and concerns. Regarding chemotherapy, topics such as (the practical consequences of) side effects, hygienic related measures, handling things at home, (a picture of) the oncology outpatient clinic, and the healthcare providers that will be present were integrated.

#### Question Prompt Sheet

A QPS consists of either a structured list of designed questions [7,39] or a blank sheet [38] on which patients can formulate their questions for their healthcare provider. Usually, patients receive a QPS before their consultation to read through and determine questions that they would like to ask. It is assumed that the use of a QPS increases the provision of personally relevant information, as patients acquire information that is tailored to their needs [40]. When the QPS is endorsed by the caregiver and introduced with clear instructions, the QPS enhances patient question asking and participation [41,42].

The QPS that was part of the brochure “Talking about chemotherapy” was used in PatientVOICE. It consists of 17 different statements about the treatment, emotions, sexuality, and coping with side effects and disease [7]. The QPS was adapted according to patients’ and nurses’ recommendations in the needs assessment and integrated in PatientVOICE.

#### Video Modeling

Video modeling is a technique that demonstrates “best practices” to patients by preparing patients for procedures, providing information, and demonstrating coping strategies or self-care behaviors. Research shows that (online) video modeling can facilitate patient understanding, improve self-care, and increase patient centeredness [43,44]. In addition, Kinnane and Thompson showed that the inclusion of a video to patient education surrounding chemotherapy improved information recall and the reporting of treatment-related symptoms [45]. In accordance with the self-management education theory of Lorig and Holman, the use of video examples appears to provide patients with the tools and (communication) skills to solve, handle, or act during certain situations [46].

In PatientVOICE, the video fragments that are used were developed for another intervention aiming to support communication between patients with malignant lymphoma in their communication with their healthcare provider [32]. The scripts were developed based on personal stories, needs, and the preferences of patients with cancer. Short video fragments about preparing for the consultation, expressing needs and concerns, and consultation audio-recordings were integrated.

#### Consultation Audio Recordings

Providing patients with an audio recording of their own consultation can be effective in improving recall [47-50]. Consultation recordings can increase understanding and comprehension, reduce the anxiety related to forgetting or not hearing important information, and facilitate communication with family members [47]. Providing consultation recordings might even enhance patients’ participation during subsequent oncology consultations [48].

An audio facility was built into PatientVOICE to upload audio files and to play the audio.

### Step 4: Program Development

**Figure 1:**
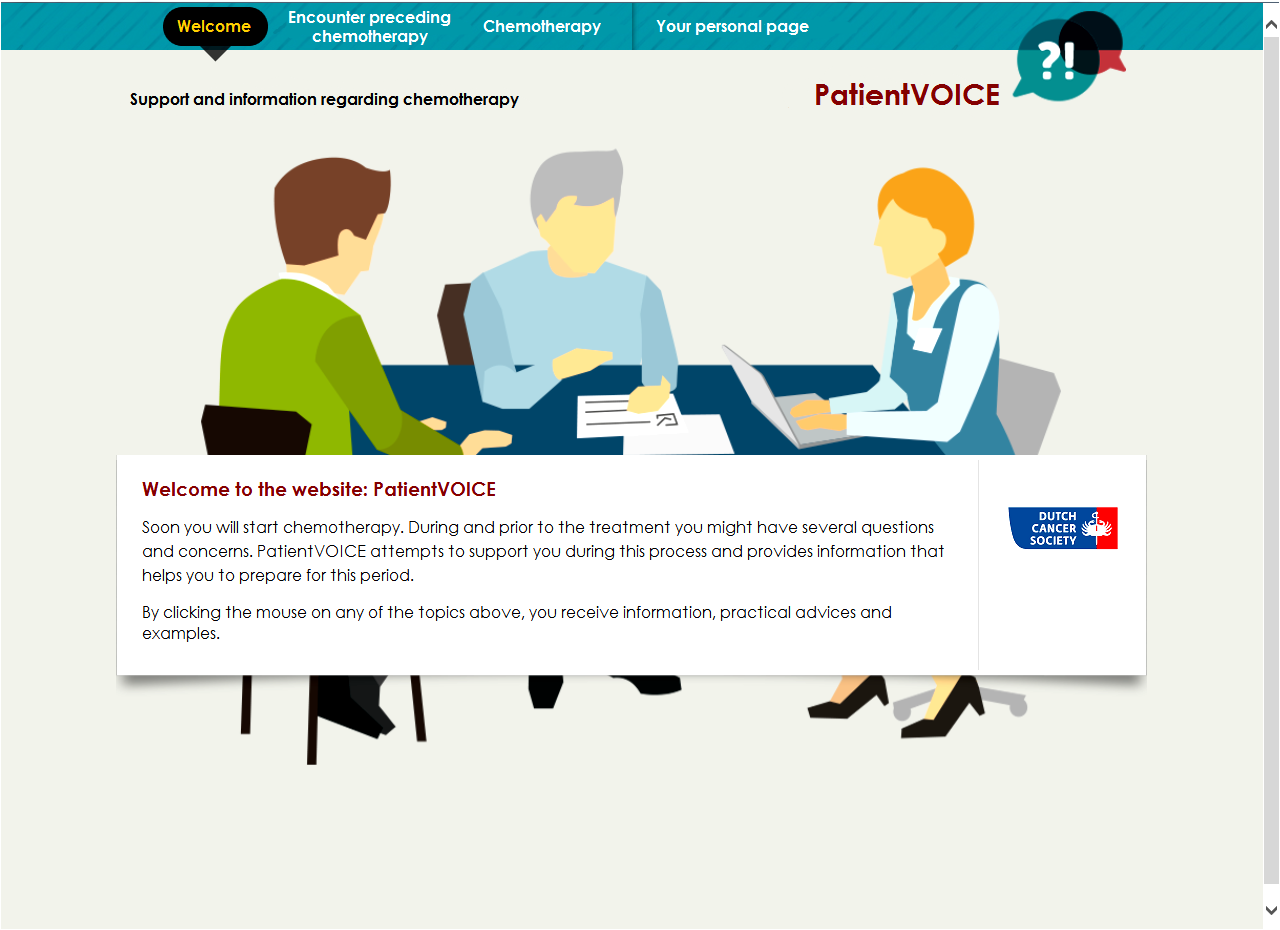
Screenshot of the homepage of PatientVOICE.

Using the outcomes from steps 1 to 3 of the intervention mapping framework, a prototype of PatientVOICE was developed (Figure 1). PatientVOICE contains a section with information on the nursing encounter preceding chemotherapy, including the QPS and video fragments, a section with information about chemotherapy, and a section that consists of a secured personal page for patients that includes the QPS and audio facility (Table 2). These sections focus mainly on the pre-counseling preparatory information. To assure privacy, the personal page is only accessible with a personal, secured login code.

**Table 2:** Overview of the content and techniques of the PatientVOICE website.

Section	Content	Technique
Welcome page	Aim and introduction of the website	N/A
Encounter preceding chemotherapy	Structure of the encounter	Pre-counseling preparatory information
	Preparation of the encounter	Pre-counseling preparatory information
		Question prompt sheet^a^
		Video modeling^b^
	Take someone to the encounter with you	Pre-counseling preparatory information
	Express your needs and concerns	Pre-counseling preparatory information
		Video modeling^c^
	Audio recording	Pre-counseling preparatory information
		Video modeling^d^
Chemotherapy	What is chemotherapy?	Pre-counseling preparatory information
	Oncology outpatient clinic	Pre-counseling preparatory information
	What providers are present at the day care setting?	Pre-counseling preparatory information
	Side effects	Pre-counseling preparatory information
	Practical measures at home	Pre-counseling preparatory information
	How do I tell it to…	Pre-counseling preparatory information
	Contacting the hospital	Pre-counseling preparatory information
	Help in making decisions	Pre-counseling preparatory information
	Useful websites	Pre-counseling preparatory information
Your personal page	Your consultation	Audio recording^e^
	Your notes	N/A
	Your questionnaire	Question prompt sheet^f^

^a^A list of 15 statements that a patient can indicate which topics he or she would like to discuss during the encounter.

^b^A video fragment in which a patient and spouse can give advice on preparing for the encounter and asking questions.

^c^A video fragment in which a patient and spouse can express their concerns and what they discussed with their healthcare provider.

^d^A video fragment showing a patient talking about their experiences with recording encounters on audio.

^e^An audio file that can be uploaded to the patient’s account. Patients have the option of listening back to their encounter.

^f^In your personal page, the question prompt sheet (QPS) can be saved.

The usability tests of the prototype indicated that participants and software experts were positive about the design and accessibility of the website, and they thought it was clear and complete. The information was understandable and plain. Some textual changes had to be made, the introduction of the QPS and the QPS itself were adapted (eg, the order of the words), and some extra options on the website were necessary to improve the navigation on the website (eg, the buttons should be bigger so that it is more clear that you can click on them). In addition, some adaptations had to be made to the layout and symbols.

The name of the website, PatientVOICE, had to be changed because patients did not understand that the name referred to chemotherapy or to the nursing encounter preceding chemotherapy; they preferred a Dutch title (‘Chemowijzer’).

Furthermore, participants navigated easily through the website. The more experience participants had with a computer or the Internet the better they were at navigating through the website. Some functionality needed to be changed, such as buttons to go to the top of the page and to return to a previous page in the QPS or to return to the website when a new page was opened. Participants with more experience were able to log in successfully, whereas participants with less experience could benefit from extra instructions about the login process. A suggestion was made to provide information about the login process by means of an instruction video, which was added.

The information about the audio recording should be somewhat clearer and more functionality was added to the audio player; having only “play” and “stop” buttons was not sufficient and functions for pausing, fast forward, and play back were added.

### Step 5: Adoption and Implementation

Healthcare providers (eg, nurses, assistants, team heads) of 4 hospitals were involved in the development of the intervention (eg, the needs assessment). After a kick-off meeting at the research institute, 2 meetings per hospital were held to discuss the content and logistics of the study and its implementation, as well as to create support for the intervention. These hospitals were very positive about the development of PatientVOICE and willing to implement the intervention.

### Step 6: Evaluation

A cross-sectional design will be used to evaluate the perceived usefulness and usability of PatientVOICE via an online questionnaire among older patients with cancer (65 years or older) who are receiving chemotherapy or have received chemotherapy in the preceding 5 years. The questionnaire will assess sociodemographics, type of cancer and chemotherapy, and the extent of their Internet and computer use. For the different sections of the website, patients will be asked whether they find the sections user-friendly, measured with the 10-item System Usability Scale [51] (ie, useful, easy to understand, helpful, reliable, reassuring, upsetting, confusing, timely, too elaborate, and complete [52]). Satisfaction with emotional support will be assessed using the Website Satisfaction Scale [53]. Regarding the QPS, patients will be asked to indicate whether it was useful, redundant, easy to complete, and whether it helped them to ask questions in the nursing encounter. Statements are also formulated about whether the video fragments were useful, redundant, realistic, gave a good example of communicating with a nurse, touched the patient emotionally, and whether patients recognized themselves in the patient in the video fragments. Regarding the audio facility, patients will be asked about the usefulness, helpfulness, and value of an audio recording and whether it helped them to recall what awaits them.

## Discussion

### Principal Findings

This paper outlined the development of the online tool PatientVOICE. By using the intervention mapping framework, we aimed to make a useful and effective intervention for older patients with cancer scheduled for a nursing encounter preceding chemotherapy. As patients and healthcare providers were involved from an early stage and at several moments in the developmental process, we attempted to take patients’ needs into account and to offer a user-friendly website for our target group. In our study, 8 older people performed the usability test. This number of assessors is sufficient to indicate 80% of the usability difficulties [54]. Since we combined patients’ and nurses’ input with empirical supported strategies to develop this intervention, we expect that the intervention not only matches with the needs of the target group, but that PatientVOICE also comprises the main factors on which to intervene, so that the intervention will be effective. In addition, we have engaged hospitals to create a support base for the intervention and expect that implementation of the intervention will go as planned when PatientVOICE is available as a public accessible website after evaluation.

Following and completing the steps of the intervention mapping framework went without difficulty. Recruiting patients and elderly for the interviews and usability tests went as planned. Our study confirms that the framework is well applicable for developing eHealth interventions. The intervention mapping framework does not impose guidelines on how to involve patients. We decided to conduct interviews with patients and to follow the “think-aloud procedure”, but other methods for patient involvement are available (eg, literature review on patients’ needs, focus groups, using questionnaires to measure needs, and involving patients as a research partner) and might have led to other outcomes.

By using the intervention mapping framework we comply with the recommendations of several parties (eg, patients, politicians, clinicians, and research funds) to increase patient participation in healthcare-related research. It is important to empower laypersons (eg, patients) in research, which is mainly expert-driven, and to improve validity, feasibility and dissemination of the research or intervention. However, the effectiveness of patient participation in research is not yet demonstrated because of the heterogeneity in methodologies used and the reporting of studies [55].

After the evaluation of PatientVOICE, we will be able to conclude whether we developed a useful and user-friendly intervention. To gain insight into the effectiveness of PatientVOICE regarding improving patients’ recall and participation in the nursing encounter, further research is needed. It has already been shown that implementation of the brochure “Talking about chemotherapy”, which contains information about the nursing consultation and the QPS, was effective. It was found that nurses talked more about realistic expectations, the amount of information was reduced in concordance with patients’ needs, and patients asked more questions during the encounters [7]. We expect that PatientVOICE will induce similar results since the brochure is the base of the website. Although patients’ recall was hardly improved in the prior study, we assume that this intervention will lead to better recall of information because of the opportunity to listen back to the audio recording of the conversation [47-50].

### Strengths and Limitations

One of the strengths in developing PatientVOICE was that we used intervention mapping which provided a systematic, clear, and workable structure. However, this framework was not applied in the proposed linear way. Especially when developing eHealth interventions, which so often remain unused, the development process benefits from starting with the adoption and implementation step as early as possible. An investigation of expected implementation barriers and facilitators among all stakeholders (in terms of organizational, financial, motivational, privacy and skills issues) at an early stage could enhance the acceptability and use of the intervention in the future. The interviews with the patients and the nurses about their needs and experiences provided valuable input for the content of the intervention. However, there are also some limitations. The needs assessment aimed to assess the patients’ needs regarding (preparing for) the consultation and to evaluate the brochure. Little attention was paid to patients’ needs regarding an online tool, what they would expect from such a website, and whether they would appreciate this kind of tool. Our interviews with nurses did show that some of them expected great benefit from an online version of our previously developed brochure, especially given the added facilities like listening back to one’s own audio recordings. Others advised us to continue to offer patients both options: the paper brochure as well as an online version. A recent study indicated that older cancer patients appreciated Web-based tools with information about cancer [26]. However, the latter study also indicated that within the group of older patients it is important to remain attentive to potential age-related problems such as cognitive and functional decline and navigation difficulties. Furthermore, nurses did not test the usability of the website, which might have indicated more points of improvement. Since nurses were not the target group of PatientVOICE, we did not ask their opinion.

### Conclusions

This article outlined the development of an online preparatory tool for patients scheduled for educational nursing encounters preceding chemotherapy, by following the consecutive steps of the intervention mapping framework. Patients (ie, the target group) and nurses were involved during several steps, as well as an examination of the theoretical literature to develop an effective and solid intervention that corresponds with patients’ needs and intervenes on determinants to change behavior. The evaluation of the intervention will give insight into the utility and usability of the intervention.

## Appendix

Multimedia Appendix 1 Content of the brochure "Talking about chemotherapy".

## Abbreviations

eHealth: electronic health
QPS: question prompt sheet
