# Supplementary material for: PatientVOICE: Development of a Preparatory, Pre-Chemotherapy Online Communication Tool for Older Patients With Cancer
Source: JMIR Res Protoc. 2017 May 10;6(5):e85. doi: 10.2196/resprot.6979 (PMC5443916; doi:10.2196/resprot.6979)
Supplement: Multimedia Appendix 1 [file resprot_v6i5e85_app1.pdf]

1. Introduction of brochure
Explanation of the aim of the brochure:

Soon you will have an encounter with a nurse about the treatment with chemotherapy. This brochure provides an overview of how this conversation will look like. You can use this brochure to prepare yourself for the encounter and to think for yourself what topics you would like to discuss with the nurse.

2. Structure of the encounter
The structure of the encounter has been provided:

Part 1 ± 25 minutes
Introduction
Information on chemotherapy and side effects

Pause ± 10 minutes
Coffee or tea

Part 2 ± 25 minutes
Discussion of topics that are important to you
Summary and closure

3. Description of the components of the encounter
Explanation of what kind of information the nurse will provide and what topics will be discussed in the parts 'Introduction', 'Information on chemotherapy', 'Information on side effects', 'Pause with coffee or tea' and 'Summary and closure'.

4. Discussion of topics that are important to you
In the part 'Discussion of topics that are important to you', patients can fill in the QPS. This QPS contains 17 statements on which a patient can indicate which topics he or she would like to discuss during the encounter. Examples:

I would like to discuss the following topics:

- *The aim of the treatment with chemotherapy*
- *The practical implications of the treatment for my daily life (e.g. household or hobbies).*
- *How to get in touch with fellow-sufferers*

After the QPS, there is an empty page on which patients can write down what they want to discuss exactly regarding the topics of the QPS and they can note other questions.
